# Influence of tumor size on oncological outcomes of pathological T3aN0M0 renal cell carcinoma treated by radical nephrectomy

**DOI:** 10.1371/journal.pone.0173953

**Published:** 2017-03-13

**Authors:** Luyao Chen, Xin Ma, Hongzhao Li, Liangyou Gu, Xintao Li, Yu Gao, Yongpeng Xie, Xu Zhang

**Affiliations:** 1 State Key Laboratory of Kidney Diseases, Department of Urology, Chinese PLA General Hospital, Beijing, China; 2 Medical School, Nankai University, Tianjin, China; Seconda Universita degli Studi di Napoli, ITALY

## Abstract

**Objective:**

To evaluate the prognostic significance of tumor size in pathological T3aN0M0 renal cell carcinoma (RCC) treated by radical nephrectomy.

**Materials and methods:**

Patients who underwent radical nephrectomy for sporadic RCC with pathological T3aN0M0 RCC at our institution between January 2006 and June 2015 were identified. The entire cohort was divided into two groups according to the cutoff of tumor size obtained from receiver operating characteristic (ROC) curve. Clinicopathological variables were retrospectively collected and compared. Kaplan-Meier analysis and multivariate Cox regression were conducted to evaluate the effect of tumor size on survival outcomes.

**Results:**

163 pT3aN0M0 RCC patients were included with a median follow-up period of 31 months. The optimal cutoff for tumor size was 7 cm according to the ROC curve. 90 cases (55.2%) presented tumors which measured 7 cm or less, and 73 cases (44.8%) showed tumor size greater than 7 cm. Patients with larger tumors tended to exhibit higher rates of symptoms and higher Fuhrman grades; they also indicated more necrosis features, and were more likely to invade the collecting system and renal vein. Compared with patients who exhibited tumor size of≤7 cm, those with tumor size>7 cm were associated with shorter estimated five-year cancer-specific survival (CSS, 46.6% versus 75.0%, *P* = 0.003) and five-year recurrence-free survival (RFS, 35.6% versus 62.7%, *P* = 0.011). Multivariate Cox analysis revealed that tumor size was retained as an independent factor for CSS (HR = 2.506, 95% CI 1.169–5.373, *P* = 0.018).

**Conclusions:**

The tumor size significantly affected the survival outcomes of pT3aN0M0 RCC treated by radical nephrectomy, and a cutoff size of 7 cm can help enhance the prognostic discrimination. Thus, the tumor size may be considered in the future TNM classification of stage pT3a.

## Introduction

Renal cell carcinoma (RCC), which is the third most common urologic tumor, accounts for approximately 3% of all reported human cancers worldwide [1]. With approximately 20%–30% of patient relapse after surgical resection [2], RCC patients should be closely followed up and stratified into categories with different recurrence and survival risks. The currently most useful determinant of RCC classification is the tumor, node, and metastasis (TNM) staging system [3], which provides critical prognostic and therapeutic information for patients. This golden standard system has been revised in recent decades to improve its prognostic accuracy and predictive ability [4, 5]. According to the latest AJCC 2010 TNM system[6], pathologic stage T1 and T2 RCC are classified depending solely on tumor size (≤7 cm for T1 and >7 cm for T2), whereas T3a is defined on the basis of anatomic tumor extension including vein or fat invasion, regardless of tumor size. Consequently, the neglected results in small and large masses are classified together, which may indicate further T3a classification modification.

The tumor size has been demonstrated as a very important prognostic factor among RCC patients [7–9]; however, the prognostic effect of tumor size in stage T3a has attracted relatively minimal attention in past studies. In 2007, Lam et al. [10] performed a retrospective analysis of 623 T3a RCC cases and concluded that the tumor size is an important predictor of cancer-specific outcomes among T3a RCC patients with fat invasion alone. Recently Suer et al. [11] also found that pT3a tumors larger than 7 cm demonstrated the worse prognosis compared to other smaller tumors after a retrospectively review of 338 consecutive patients with pT1-3aN0M0 RCC, involving 63 pT3a tumors,. These interesting findings indicate that the prognostic role of tumor size may apply to not only low (T1 and T2) but also high (T3a) tumor stages.

In the present study, we retrospectively analyzed the records of pT3aN0M0 RCC patients in the database of our institution. We also evaluated the significance of tumor size by assessing its effect on patient survival outcomes and its association with other clinicopathological factors.

## Materials and methods

### Patient selection and data collection

After obtaining approval from the ethical committee of the Chinese PLA General Hospital, we retrospectively analyzed the database of 4,520 consecutive patients surgically treated for sporadic RCC in our institution between January 2006 and June 2015. According to the 2010 AJCC TNM system, we identified 172 unilateral stage pT3aN0M0 RCC patients who underwent radical nephrectomy (through repetitive reviews by two independent pathologists). Of these patients, 9 were lost in follow-ups, and the remaining 163 patients were included in the present study. This research was conducted in accordance with the approved guidelines, and written informed consent was obtained from all included patients.

Chest x-ray and abdominal CT/MRI were used for the preoperative clinical staging of the patients. Bone scan and brain imaging were performed when indicated by corresponding symptoms. After surgical resection, all pathological specimens were reviewed internally by our institution’s department of pathology. Postoperative pathologic tumor stage and grade were determined in accordance with the seventh AJCC TNM staging system [6] and Fuhrman grading system [12], respectively. Histological subtypes of RCC were assigned in accordance with the WHO classification system [13]. We evaluated the following clinical and pathologic features: gender, age at surgery, symptoms at presentation (hematuria, osphyalgia, abdominal mass, etc.), tumor location, tumor size, histological subtype, Fuhrman nuclear grade, presence of necrosis, sarcomatoid differentiation, collecting system invasion, perirenal/sinus fat invasion, and renal vein involvement for every patients.

Postsurgical follow-ups were performed in all patients in accordance with our institution’s protocol. Physical examination, ultrasound and CT scan were accomplished every 3 or 6 months for the first 2 years. After the second year, physical and imaging examinations were conducted annually. Further investigations were individually adjusted as indicated by the clinical symptoms. Disease and vital statuses were recorded regularly through clinic follow-ups or telephone interviews. The survival outcomes in our study were cancer-specific survival (CSS) and recurrence-free survival (RFS). Survival time was calculated from the surgery to the recurrence dates, the RCC death, or the last follow-up.

### Statistical analysis

Continuous variables were reported as mean value ± standard deviation (SD) or median value and range/interquartile range (IQR), as appropriate. Student *t*-test or Mann–Whitney U test were applied to compare continuous variables, whereas Pearson chi-square or Fisher’s exact test were used to compare categorical variables. The optimal cutoff value of tumor size in prediction of survival outcomes was determined by Receiver Operating Characteristic (ROC) curve analysis. The longest tumor diameter was selected as the independent variable and the survival outcome (dead/alive for CSS and progressed/disease free for RFS) was selected as the dependent variable. Besides, the area under the ROC curve (AUC) was used to calculate discrimination ability. The CSS and RFS were estimated using the Kaplan–Meier method, and the log-rank test was applied to compare survival curves. Univariate and multivariate Cox proportional hazard regression models were used to assess the prognostic effects of clinical and pathological factors. The associations between the variables studied and the survival outcomes were presented as hazard ratios (HRs) with corresponding 95% confidence intervals (CIs). All analyses were performed using SPSS 19.0 (SPSS Inc. Chicago, IL, USA). All comparisons were two-sided and considered statistically significant at *P*<0.05.

## Results

### Patient characteristics

The clinical and pathological characteristics of 163 T3aN0M0 RCC patients who underwent radical nephrectomy are summarized in Table 1. This study included 123 men (75.5%) and 40 (24.5%) women. The mean (± SD) age was 56.5±12.4 years. Symptoms were present during the diagnosis in 76 patients (46.6% of the cases). The mean (± SD) and median (IQR) pathological tumor dimensions were 6.8±3.5 and 6.0 (4.0−9.0) cm, respectively. The pathological examination indicated clear cell RCC as the most common subtype (82.8%); moreover, 83 and 55 patients were classified with low (G1/G2) and high grades (G3/G4),correspondingly, according to the Furhman grading system (25 patients without data). Furthermore, 53 (32.5%) and 9 specimens (5.5%) revealed tumor necrosis and sarcomatoid differentiation, respectively. Invasion into collecting system was recorded in 40 patients (24.5%), fat invasion was documented in 90 patients including 79 (48.5%) with perinephric fat and 11 with sinus fat invasions (6.7%), and 87 cases (53.4%) showed renal vein thrombus. The mean (± SD) and median (range) follow-ups of 163 T3aN0M0 RCC patients were 34.4(±22.9) and 31.0 (3.4−109.7) months, respectively. During the follow-up period, a total of 68 patients (41.7%) presented disease recurrence (local or distant), and 44 patients (27.0%) died of RCC.

**Table 1:** Clinicopathological characteristics of 163 patients with pT3aN0M0 RCC and subgroup comparison of variables according to the tumor size (cutoff of 7cm).

	Total	≤7cm	>7cm	*P* value
Variable	n = 163	n = 90	n = 73	
**Male (%)**	123(75.5)	67(74.4)	56(76.7)	0.738
**Age (mean±SD)**	56.5±12.4	56.9±12.8	56.1±12.0	0.667
**Presenting symptom (%)**	76(46.6)	32(35.6)	44(60.3)	0.002
**Tumor location (%)**				0.077
Left	88(54.0)	43(47.8)	45(61.6)	
Right	75(46.0)	47(52.2)	28(38.4)	
**Tumor size (mean±SD)**	6.8±3.5	4.3±1.3	9.8±3.1	<0.001
**Histological subtype (%)**				0.740
Clear cell	135(82.8)	74 (82.2)	61(83.6)	
Papillary	8(4.9)	6(6.7)	2(2.7)	
Chromphobe	2(1.2)	1(1.1)	1(1.4)	
Collecting duct	3(1.8)	2(2.2)	1(1.4)	
Unclassified	15(9.2)	7(7.8)	8(10.9)	
Fuhrman grade* **(%)**				0.009
Low:1,2	83(60.1)	52(70.3)	31(48.4)	
High:3,4	55(39.9)	22(29.7)	33(51.6)	
**Tumor necrosis (%)**	53(32.5)	21(23.3)	32(43.8)	0.005
**Sarcomatoid differentiation (%)**	9(5.5)	6(6.7)	3(4.1)	0.732
**Collecting system invasion (%)**	40(24.5)	16(17.8)	24(32.9)	0.026
**Perirenal fat invasion (%)**	79(48.5)	51(56.7)	28(38.4)	0.020
**Sinus fat invasion (%)**	11(6.7)	5(5.6)	6(8.2)	0.543
**Renal vein invasion (%)**	87(53.4)	40(44.4)	47(64.4)	0.011

*25 patients without data.

### Tumor size and clinicopathological features

**Fig 1:**
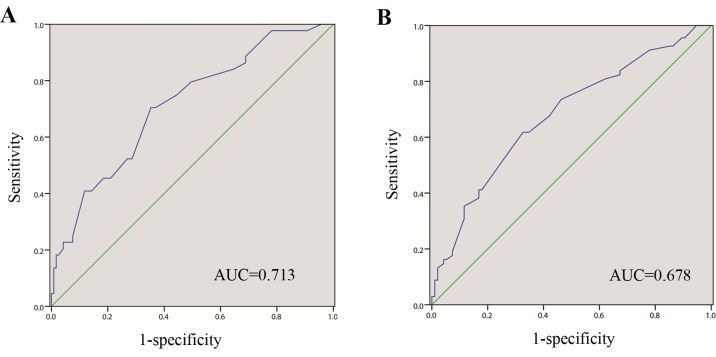
Receiver operating characteristic curves were used to identify the optimal cutoff value of tumor size predicting survival. (A) cancer-specific survival and (B) recurrence-free survival.

ROC curves were constructed to determine the appropriate cutoff point of tumor size (Fig 1). The most discriminative tumor size cutoff value of 7 cm was selected for both CSS (sensitivity = 70.5%, specificity = 64.7%; AUC = 0.713, 95% CI 0.625–0.801, P<0.001) and RFS (sensitivity = 61.8%, specificity = 67.4%; AUC = 0.678, 95% CI 0.594–0.762, P<0.001). The entire cohort was then divided into two groups according to tumor dimensions (group A, n = 90, tumor size≤7 cm; group B, n = 73, tumor size>7 cm). The clinicopathological features are compared in Table 1. The following variables: gender (P = 0.738), age (P = 0.667), tumor location (P = 0.077), histological subtype (P = 0.740), sarcomatoid differentiation (P = 0.732), and sinus fat invasion (P = 0.534) were similar between the two groups. By contrast, a statistically significant difference was found in the mean tumor size (4.3±1.3 cm versus 9.8±3.1 cm, P<0.001). Patients with larger tumors presented higher rates of symptoms at diagnosis (60.3% versus 35.6%, P = 0.002) and low proportions of perirenal fat invasion (38.4% versus 56.7%, P = 0.020). Furthermore, group B was significantly more likely to achieve higher Fuhrman grades (33/22 versus 31/52, P = 0.009), and necrosis evidence (43.8% versus 23.3%, P = 0.005). Significant differences regarding invasion of collecting system (32.9% versus 17.8%, P = 0.026) and renal vein (64.4% versus 44.4%, P = 0.011) were also observed between the two groups.

### Survival analysis

**Fig 2:**
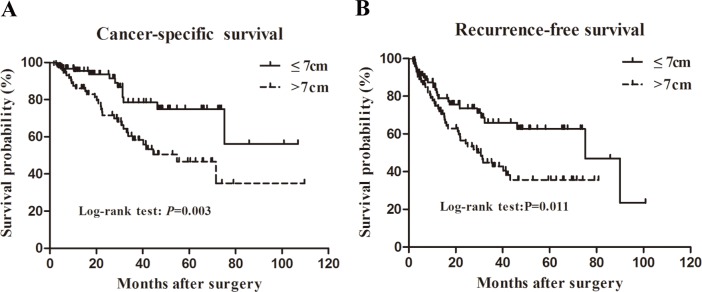
Kaplan–Meier analysis of survival stratified by tumor size with a cutoff of 7 cm. (A) cancer-specific survival and (B) recurrence-free survival.

The CSS curves by the Kaplan–Meier survival analysis are shown in Fig 2. Patients with tumor size of >7 cm experienced significantly worse estimated five-year CSS rates than those with tumor size of ≤7 cm (46.6% versus 75.0%, *P* = 0.003). Similarly, the estimated five-year RFS rates in group B were significantly lower than those in group A (35.6% versus 62.7%, *P* = 0.011, Fig 2). According to the univariate analysis, tumor size (*P* = 0.003), histological subtype (*P* = 0.035), Fuhrman grade (*P* = 0.002) and sarcomatoid differentiation (*P* = 0.037) appeared as significant prognostic factors for CSS, in contrast to other variables including gender, age, symptom, location, necrosis, invasion of collecting system, fat, and vein (Table 2). To further identify the independent prognostic factors, multivariate analysis with Cox proportional hazard ratio model was performed. The results confirmed tumor size (subgrouped by 7 cm), together with Fuhrman grade, as an independent prognostic factor for CSS (HR = 2.506, 95% CI 1.169–5.373, *P* = 0.018). For RFS (Table 3), univariate analysis revealed that tumor size (*P* = 0.011), Fuhrman grade (*P*<0.001), tumor necrosis (*P* = 0.005), sarcomatoid differentiation (*P* = 0.004) and collecting system invasion (*P* = 0.005) were significant predictors. However, the tumor size failed to be an independent prognostic factor (HR = 1.510, 95% CI 0.850–2.682, *P* = 0.160) when these variables were included in the multivariate Cox analysis, and only the Fuhrman grade remained as a significant prognostic predictor for RFS (HR = 2.191, 95% CI 1.212–3.961, *P* = 0.009).

**Table 2:** Univariate and multivariate Cox regression analysis of clinicaopathological variables for cancer-specific survival.

		Univariate			Multivariate	
Variable	HR	95%CI	*P* value	HR	95%CI	*P* value
Sex (male vs female)	1.037	0.511–2.102	0.921			
Age (continuous)	0.981	0.958–1.005	0.123			
Presenting symptom	1.391	0.763–2.535	0.281			
Tumor location (left vs right)	1.309	0.717–2.388	0.380			
Tumor size (>7cm vs ≤7cm)	2.567	1.341–4.917	0.003	2.506	1.169–5.373	0.018
Histological subtype (non-ccRCC vs ccRCC)	2.086	1.052–4.137	0.035	2.408	0.857–6.763	0.095
Fuhrman grade (high vs low)	2.960	1.493–5.871	0.002	2.452	1.218–4.937	0.012
Tumor necrosis	1.629	0.867–3.061	0.129			
Sarcomatoid differentiation	2.707	1.062–6.899	0.037	1.432	0.375–5.464	0.599
Collecting system invasion	1.813	0.970–3.388	0.062			
Perirenal fat invasion	1.295	0.715–2.344	0.393			
Sinus fat invasion	1.509	0.465–4.904	0.493			
Renal vein invasion	1.070	0.586–1.954	0.827			

Abbreviation: HR, hazard ratio; CI, confidence interval; ccRCC, clear cell renal cell carcinoma.

**Table 3:** Univariate and multivariate Cox regression analysis of clinicaopathological variables for recurrence-free survival.

		Univariate			Multivariate	
Variable	HR	95%CI	P value	HR	95%CI	P value
Sex (male vs female)	0.942	0.530–1.676	0.840			
Age (continuous)	0.981	0.962–1.001	0.058			
Presenting symptom	1.415	0.869–2.305	0.163			
Tumor location (left vs right)	1.214	0.749–1.969	0.431			
Tumor size (>7cm vs ≤7cm)	1.964	1.195–3.227	0.011	1.510	0.850–2.682	0.160
Histological subtype (non-ccRCC vs ccRCC)	1.682	0.942–3.003	0.079			
Fuhrman grade (high vs low)	2.837	1.638–4.914	0.000	2.191	1.212–3.961	0.009
Tumor necrosis	2.025	1.237–3.317	0.005	1.274	0.718–2.261	0.407
Sarcomatoid differentiation	3.158	1.434–6.953	0.004	1.926	0.664–5.588	0.228
Collecting system invasion	2.051	1.341–3.388	0.005	1.473	0.810–2.679	0.204
Perirenal fat invasion	1.197	0.740–1.934	0.464			
Sinus fat invasion	1.753	0.755–4.073	0.192			
Renal vein invasion	0.970	0.599–1.570	0.900			

Abbreviation: HR, hazard ratio; CI, confidence interval; ccRCC, clear cell renal cell carcinoma.

## Discussion

Considering that the TNM staging system has become the most frequent classification in clinical prognostic prediction, this stratification should be continuously revised and modified as new data are collected to remain up to date with the modern demands of evidence-based practice [14]. According to the latest TNM system edition [6], in contrast to tumors confined to kidney (T1 and T2), T3a is defined on the basis of the anatomic tumor extension regardless of tumor size. However, whether tumors in the same stage (T3a) with significantly different sizes exhibit the same prognosis remains unclear.

The entire cohort of the present study, including 163 pT3aN0M0 RCC patients, was divided into two groups with optimal tumor size cutoff of 7 cm. Patients with larger tumors tended to achieve higher rates of symptoms during diagnosis and higher Fuhrman grades; they also showed tumors with more necrosis features and were more likely to invade the collecting system and renal vein. However, there was no difference of sinus fat invasion between these two groups, which may attribute to the small percentage of sinus fat invasion in our data (6.7%). The survival analysis further showed that sinus fat infiltration did not influence the prognosis of pT3aN0M0 RCC, whereas this finding was different from the conclusion from a recent meta-analysis [15]. Hence, more studies with larger samples are required in future to answer this conflicting question. Compared with those with tumor size≤7 cm, Kaplan–Meier survival analysis demonstrated that the tumor size of >7 cm was associated with worse estimated five-year CSS and RFS rates. After adjusting for other variables, further multivariate Cox hazard ratio analysis showed that tumor size remained as an independent predictor for CSS with a 2.51-fold higher risk of dying in tumor size of >7 cm. However, the tumor size failed to be an independent prognostic factor for RFS, and the Fuhrman grade was the only parameter that can independently influence both CSS and RFS in pT3aN0M0 RCC.

Several groups have recently evaluated the role of tumor size in T3a RCC, and they have obtained inconsistent results. Siemer et al. [16] analyzed 237 patients with perirenal fat invasion and identified an ideal tumor size cutoff of 7 cm to significantly distinguish the prognosis of different groups. In addition, they suggested a modified T stage classification, in which T1 should incorporate T3a with tumor size of ≤7 cm, and T2 should be modified to include all T3a with tumor size of >7 cm. However, these findings were not in accordance with another multicenter study by Lam et al. [10], who found that the survival of patients with T2 was significantly superior to T3a>7 cm, whereas T3a≤7 cm did not differ from T2 and T3a>7 cm experienced survival outcomes similar to stage T3b. Similarly, after retrospectively analyzing 77 T3aN0M0 RCC patients, Yoo et al. [17] concluded that tumor size was the strongest CSS prognostic factor, and suggested that it should be included in the T3a staging. Gofrit et al. [18] also reported that stage T3a RCC was an inhomogeneous group wherein small tumors and excellent prognosis along with large tumors and poor prognosis were associated. However, the abovementioned studies were based on the sixth edition of the TNM classification, in which the ipsilateral adrenal gland and renal vein invasion was classified as stage pT3a and pT3b, respectively.

To our knowledge, limited literature has investigated the influence of tumor size on T3a RCC according to the latest edition (seventh edition). Schiavina et al. [19] evaluated 185 T3a RCC patients, involving 29 positive lymph nodes, who underwent surgery. They divided these patients into two subgroups according to tumor size with an 8 cm cutoff (slightly higher than our study). Consistent with our results, larger tumors achieved higher rates of symptomatic cases and necrosis features. In multivariate analysis, patients with larger tumors showed an independent 3.65-fold higher risk of cancer-specific death compared with smaller tumors. Chevinsky et al. [20] assessed the increased recurrence risk by the tumor size of 326 patients with pT3a RCC (65% were 7 cm or less, and 35% were greater than 7 cm). Their results demonstrated that patients with increasing tumor size indicated significantly greater disease recurrence risks. Another multicenter study by Brookman-May et al. [21] also discussed the prognostic role of tumor size in pT3aN0M0 RCC patients. They concluded that tumor size can significantly influence cancer-specific mortality (in which a 1 cm increase was associated with a 7% increase in cancer-specific mortality), and a cutoff of 7 cm was the optimal and practical means to stratify the significantly different prognoses among pT3a RCC patients. These findings, are consistent with ours in supporting the consideration of including tumor size with a useful and practical cutoff in future TNM staging systems to help improve the prognostic discrimination for patients with stage pT3a tumors.

Our study includes several limitations that need to be acknowledged. The retrospective design of our work, as well as its restriction to a relatively low number of enrolled patients are the main drawbacks of this study. Although we initially assessed the influence of tumor size on both CSS and RFS in T3aN0M0 RCC, the median follow-up for our patients was slightly shorter than those of some previous studies. Besides, details regarding comorbidity, laboratory parameters, and recurrent disease treatment were unavailable for all patients; hence, these factors were not analyzed. Furthermore, a number of validated molecular makers and nephrometry scoring systems were not included in our present analysis. Although the tumor size with an optimal and practical cutoff improved the prognostic discrimination of T3a staging, future external and prospective studies are evidently necessary to resolve this issue and reevaluate the current TNM system.

## Conclusions

Our findings revealed the significant influence of tumor size on the survival outcomes in pT3aN0M0 RCC patients; furthermore, a 7cm cutoff can help improve the prognostic discrimination. Thus, tumor size may be considered for inclusion in the TNM classification of stage pT3a in the future. Nevertheless, further research is needed to confirm this suggestion.

## Supporting information

S1 File approval document from ethics committee-Chinese version. (PDF)

S2 File approval document from ethics committee-English version. (PDF)

S3 File STROBE_checklist_v4_combined_PlosMedicine. (DOCX)
