# Supplementary material for: Influence of tumor size on oncological outcomes of pathological T3aN0M0 renal cell carcinoma treated by radical nephrectomy
Source: PLoS One. 2017 Mar 13;12(3):e0173953. doi: 10.1371/journal.pone.0173953 (PMC5348019; doi:10.1371/journal.pone.0173953)
Supplement: S1 File — (PDF) [file pone.0173953.s001.pdf]

解放军总医院医学伦理委员会
临床项目审批件

**项目名称**	肿瘤大小对肾细胞癌（T3aN0M0）的预后影响研究
**项目内容**	通过对我院泌尿外科 2006 年至 2015 年肾癌手术资料数据库进行回顾性分析，筛选出病理分期为 T3aN0M0 的肾癌患者资料。通过随访患者生存信息，评估肿瘤大小对该部分患者肿瘤学预后的影响。
**项目类型**	回顾性研究
**申请科室**	外科临床部泌尿外科
**申请人**	张旭
**申请日期**	2016 年 1 月 1 日

科室审核意见：

同意开展此课题项目。

科室负责人签名：

日期：2016 年 1 月 10 日

伦理委员会审核意见：

经审查，该课题项目符合医学伦理委员会相关要求，准予开展实施。

中国人民解放军总医院医学伦理委员会

盖章

日期：2016 年 2 月 1 日
