# Supplementary material for: Influence of tumor size on oncological outcomes of pathological T3aN0M0 renal cell carcinoma treated by radical nephrectomy
Source: PLoS One. 2017 Mar 13;12(3):e0173953. doi: 10.1371/journal.pone.0173953 (PMC5348019; doi:10.1371/journal.pone.0173953)
Supplement: S2 File — (PDF) [file pone.0173953.s002.pdf]

The Ethical Committee of Chinese PLA General Hospital

clinical study approval

**Project name**	Influence of Tumor Size on Survivals of Pathological T3aN0M0 Renal Cell Carcinoma
**Project content**	In the present study, we retrospectively analyzed the records of pT3aN0M0 RCC patients in the database of our institution from 2006 to 2015. We also evaluated the significance of tumor size by assessing its effect on patient survival outcomes and its association with other clinicopathological factors.
**Project style**	Retrospective study
**Department**	Department of Urology
**Applicant**	Xu Zhang
**Date**	1/1/2016

Department Opinion:

Approve this project.

Signature of director: Xu Zhang

Date: 10/1/2016

The Ethical Committee Opinion:

This project applied by Xu Zhang meets the requirement of the Ethical Committee and is approved.

Stamp

Date: 1/2/2016
